# Análisis comparativo de la actividad antimicrobiana de secreciones y excreciones larvarias de *Calliphora vicina* y *Sarconesiopsis magellanica* (Diptera: Calliphoridae)

**DOI:** 10.7705/biomedica.6067

**Published:** 2022-03-01

**Authors:** Francy Novoa-Palomares, Laura Salas-Díaz, Cindy Pérez-Téllez, Ingred Pinillos- Medina, Orlando Torres-García, Felio J. Bello

**Affiliations:** 1 Facultad de Ciencias de la Salud, Universidad Colegio Mayor de Cundinamarca, Bogotá, D.C., Colombia Universidad Colegio Mayor de Cundinamarca Facultad de Ciencias de la Salud Universidad Colegio Mayor de Cundinamarca Bogotá D.C Colombia; 2 Facultad de Medicina Veterinaria, Universidad Antonio Nariño, Bogotá, D.C., Colombia Universidad Antonio Nariño Facultad de Medicina Veterinaria Universidad Antonio Nariño Bogotá D.C Colombia; 3 Facultad de Ciencias Agropecuarias, Universidad de La Salle, Bogotá D.C., Colombia Universidad de la Salle Facultad de Ciencias Agropecuarias Universidad de La Salle Bogotá D.C Colombia

**Keywords:** dípteros, bacterias Gram positivas, bacterias Gram negativas, modalidades de secreciones y excreciones, antibacterianos, larva, Diptera, Gram-positive bacteria, Gram-negative bacteria, modalities, secretion and excretion, anti-bacterial agents, larva

## Abstract

**Introducción.:**

La creciente resistencia bacteriana a los antibióticos representa una amenaza mundial de salud pública. Las excreciones y secreciones larvarias derivadas de moscas necrófagas de la familia Calliphoridae podrían configurar una fuente promisoria para contrarrestar sus efectos.

**Objetivo.:**

Comparar la actividad antimicrobiana de las excreciones y secreciones larvarias nativas, y de las mayores y menores de 10 kDa de *Calliphora vicina* y *Sarconesiopsis magellanica* (Diptera: Calliphoridae).

**Materiales y métodos.:**

El bioensayo se hizo a partir de la técnica de turbidimetría y en el caso de las excreciones y secreciones menores de 10 kDa se determinó la concentración inhibitoria mínima (CIM).

**Resultados.:**

Las excreciones y secreciones nativas y las menores de 10 kDa de *C. vicina* y *S. magellanica*, evidenciaron una potente actividad antibacteriana contra tres cepas de *Staphylococcus aureus* y cuatro bacterias Gram negativas, siendo las menores de 10 kDa más efectivas que las nativas en las dos especies de moscas evaluadas. Además, las menores de 10 kDa presentaron la misma efectividad, aunque en las pruebas de CIM se observó que las de *S. magellanica* fueron más potentes en todas las bacterias evaluadas, excepto contra la cepa de *S. aureus* ATCC 25923. Las mayores de 10 kDa no inhibieron el crecimiento bacteriano.

**Conclusión.:**

Los resultados validaron, en general, que estas sustancias son fuente importante para el aislamiento y la caracterización de agentes antimicrobianos.

## Abstract (en)

**Introduction:**

The growing resistance to antibiotics worldwide represents a global threat to public health. The larval excretions and secretions derived from necrophagous flies from the Calliphoridae family could represent a promising source for counteracting their effects.

**Objective:**

To compare the antimicrobial activity of *Calliphora vicina* and *Sarconesiopsis magellanica* (Diptera: Calliphoridae) native excretions and secretions and those weighing more than 10 kDa and less.

**Materials and methods:**

We used the turbidimetry technique for the bioassay; we determined the minimum inhibitory concentration (MIC) for excretions and secretions weighing less than 10 kDa.

**Results:**

*Calliphora vicina* and *S. magellanica* native excretions and secretions and those weighing less than 10 kDa exhibited potent antibacterial activity against three *Staphylococcus aureus* strains and four Gram-negative bacteria; those weighing less than 10 kDa were more effective than the native ones in the two species of flies evaluated here. Furthermore, excretions and secretions weighing less than 10 kDa had the same effectiveness, except in the MIC trials where *S. magellanica* excretions and secretions weighing less than 10 kDa were more potent against all the bacteria evaluated, except for *S. aureus* ATCC 25923. Excretions and secretions weighing more than 10 kDa did not inhibit bacterial growth.

**Conclusions:**

These results potentially validate these substances as an important source for isolating and characterizing antimicrobial agents.

La familia Calliphoridae está constituida por moscas con calípteros distribuidas en todo el mundo con alrededor de 1.000 especies, de las cuales 126 se encuentran en el Neotrópico ^1^. *Calliphora vicina* (Robineau-Desvoidy) (Diptera: Calliphoridae) es una mosca sinantrópica que se ha registrado en Colombia en los departamentos de Casanare, Tolima, Santander, Caldas, Valle del Cauca, Meta y Cundinamarca, específicamente en la Sabana de Bogotá, en zonas ubicadas a 2.500 metros sobre el nivel del mar (msnm) ^2^. *Sarconesiopsis magellanica* (Le Guillou) (Diptera: Calliphoridae) es una especie hemisinantrópica que se distribuye en los departamentos de Antioquia, Boyacá, Cundinamarca y Norte de Santander, entre los 1.200 y los 3.100 msnm ^1^.

Las moscas *C. vicina* y *S. magellanica*, así como otras especies de esta familia, son de gran importancia en medicina humana y veterinaria debido a que sus larvas causan miasis en humanos y animales ^3^; además, los adultos actúan como vectores mecánicos de algunas especies de bacterias, protozoos y helmintos ^4^. Por sus hábitos necrófagos, estas moscas se utilizan en los estudios forenses para determinar el intervalo *post mortem*^5^. Asimismo, las especies de la familia Calliphoridae se han estudiado ampliamente por los efectos benéficos de las larvas en heridas de difícil cicatrización, como las úlceras diabéticas crónicas ^6^, ya que limpian las heridas infectadas y necróticas, facilitando así su cicatrización, en lo que se conoce como terapia larvaria o “biocirugía” ^6^. El proceso de curación de heridas se da a partir de tres acciones sinérgicas: el desbridamiento ^7^, la desinfección ^8^ y erradicación de biopelículas ^9^, y la estimulación del tejido de granulación en la cicatrización ^9^.

En cuanto al efecto antimicrobiano, las larvas ingieren bacterias dentro del tejido necrótico, eliminando así los microorganismos presentes ^10^; además, en sus excreciones y secreciones liberan un amplio espectro de sustancias con la capacidad de inhibir el crecimiento de bacterias Gram negativas y Gram positivas ^11^. Estos hallazgos son potencialmente útiles en el campo médico, ya que las propiedades antibacterianas de las excreciones y secreciones larvarias pueden ser una alternativa en el tratamiento y control de las enfermedades infecciosas no controladas con los antibióticos convencionales tras su uso masivo y desregulado, el cual ha propiciado el desarrollo de resistencia bacteriana asociada con gran morbilidad y mortalidad en todo el mundo ^12^.

En algunos estudios se ha demostrado la efectividad antibacteriana de las excreciones y secreciones larvarias de diferentes especies de moscas de la familia Calliphoridae, como *Lucilia sericata*^13^^,^^14^, *S. magellanica*^15^, *C. vicina*^16^, *Chrysomya putoria*^17^, *C. megacephala*^17^, *C. rufifacies*^18^ y *Cochliomya macellaria*^18^, principalmente contra las bacterias *Staphylococcus aureus* (Bacillales: Staphylococcaceae), *Pseudomonas aeruginosa* (Pseudomonadales: pseudomonadaceae) y *Escherichia coli* (Enterobacterales: enterobacteriaceae), las cuales representan un serio problema debido a que son agentes patógenos asociados frecuentemente con infecciones hospitalarias e infecciones en heridas de difícil cicatrización.

El objetivo principal de este trabajo fue comparar la actividad antimicrobiana de las excreciones y secreciones nativas, y de aquellas mayores y menores de 10 kDa, derivadas de larvas de *C. vicina* y *S. magellanica* contra cuatro bacterias Gram negativas y cuatro Gram positivas.

## Materiales y métodos

### Colonización y mantenimiento de las colonias de las moscas Calliphora vicina y Sarconesiopsis magellanica

Los especímenes adultos de ambas especies se recolectaron con una jama entomológica en el Parque Nacional Enrique Olaya Herrera de Bogotá, localizado en las coordenadas 4°37’28,2”N 74°03’56,3”W. La identificación morfológica se hizo bajo un estereoscopio con base en la clave ilustrada para la identificación de los géneros y especies de califóridos (Diptera: Calliphoridae) de Colombia ^19^.

Se contó con los permisos de recolección contemplados en la Resolución 0922 del 15 de mayo de 2017 expedida por el Ministerio de Ambiente y Desarrollo Sostenible.

En la iniciación de la colonia, los adultos de *C. vicina* y *S. magellanica* se introdujeron en jaulas entomológicas Gerber bajo condiciones controladas de laboratorio a una temperatura de 25 ± 1 °C, humedad relativa de 60 ± 5 % y fotoperiodo de 12:12; su alimentación consistió en hígado como fuente proteica y agua azucarada como fuente de carbohidratos. Los huevos se transfirieron a frascos de vidrio identificados con el nombre de cada especie, los cuales contenían 10 g de hígado de cerdo. Después de la eclosión, se agregó más hígado para permitir el desarrollo larvario y, en un tiempo aproximado de cinco días, se tomaron las larvas de tercer estadio.

### Obtención de excreciones y secreciones larvarias

En las pruebas se utilizaron 3.000 larvas de tercer estadio de *C. vicina* y *S. magellanica*, las cuales fueron inmunizadas previamente con el fin de activar el sistema inmunológico y aumentar la expresión de los componentes con actividad antibacteriana. En este proceso, se empleó una mezcla en suspensión de cuatro especies de bacterias Gram positivas (tres cepas de *S. aureus* y *S. pneumoniae*) y cuatro Gram negativas (*E. coli*, *P. aeruginosa*, *S. marcescens* y *K. pneumoniae*) con una concentración de 0,5 en la escala de McFarland durante una hora a 37 °C ^15^.

Enseguida, se procedió a la desinfección de las larvas con 0,5 % de hipoclorito durante cinco minutos, seguida de un lavado con 5 % de formaldehído por cinco minutos y, por último, tres inmersiones durante tres minutos en solución salina estéril ^15^. Posteriormente, se agregaron 100 μΙ de solución salina estéril a las larvas y se incubaron a 25 °C durante una hora para inducir las excreciones y secreciones; después, estas se extrajeron y se llevaron a tubos Eppendorf de 2 ml y se centrifugaron a 13.000g a una temperatura de 4 °C durante 10 minutos. Para eliminar posibles contaminantes, el sobrenadante se filtró a través de una membrana de 0,22 μm (Ultra CruzTM) ^15^.

### Filtración

Al cabo del proceso anterior, se tomaron 10 ml de excreciones y secreciones nativas de *C. vicina* y *S. magellanica*; luego; se filtraron usando una membrana Amicon Ultra 15^™^. Las de peso molecular de 10 kDa, se centrifugaron durante 10 minutos a 4 °C al alcanzar los 4.200*g* y se obtuvieron fracciones mayores y menores de10 kDa.

### Cuantificación de proteínas

Para esto se empleó espectrofotometría ultravioleta-visible (UV-VIS) a 280 nm en un equipo NanoDrop 2000c (Thermo Scientific^TM^) usando 2 μΙ de excreciones y secreciones nativas, y 2 μΙ de las menores de 10kDa, volúmenes que fueron previamente obtenidos en el proceso de filtración.

### Actividad antimicrobiana

*Bacterias*. Las cepas seleccionadas para evaluar la actividad antimicrobiana fueron: S. aureus ATCC 25923 (Bacillales: Staphyloccocaceae), *S. aureus* ATCC 6538, *S. aureus* ATCC 43300 (cepa resistente a la meticilina: MRSA, *methicillin-resistant* S. *aureus*), S. *pneumoniae* ATCC 6303 (Lactobacillales: Streptoccocaceae), *E. coli* ATCC 26922 (Enterobacterales: enterobacteriaceae), *P. aeruginosa* ATCC 1744 BAA (Pseudomonadales: Pseudomonadaceae), *Serratia marcescens* ATCC 13880 (Enterobacterales: Yersiniaceae) y *Klebsiella pneumoniae* ATCC 700603 (Enterobacterales: Enterobacteriaceae). Estas cepas se seleccionaron dado que en ellas no se ha estudiado ampliamente el potencial antimicrobiano de las excreciones y secreciones.

### Pruebas de inhibición de crecimiento en medio líquido mediante turbidimetría

Se evaluó el potencial antimicrobiano de las excreciones y secreciones nativas, y de las mayores o menores de 10 kDa, tanto de *C. vicina* como de *S. magellanica*. En la microplaca, se agregaron 100 μl de medio LB como control, 100 μl de medio con bacteria como control negativo y, como control positivo, se utilizaron dos antibióticos, gentamicina (10 μg/ml) para bacterias Gram negativas y estreptomicina-penicilina (10 μg - 10 UI) para bacterias Gram positivas; y se adicionaron 50 μl de cada antibiótico en 50 μl de medio con la bacteria seleccionada; para las excreciones y secreciones nativas, y las mayores o menores de10 kDa, se agregaron 50 μl de cada una a 50 μl de medio con bacteria.

Las pruebas se practicaron por triplicado, el tiempo de incubación fue de 18 horas a 37 °C y la lectura de la absorbancia se hizo a una longitud de onda de 620 nm. El porcentaje de crecimiento se determinó empleando la siguiente formula:




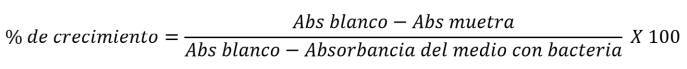




### Prueba de concentración inhibitoria mínima en medio líquido mediante turbidimetría

Para la prueba de CIM se hicieron diluciones seriadas de 1:2 a partir de la concentración de proteína obtenida de las excreciones y secreciones mayores de 10 kDa de larvas de tercer estadio. Para las de *S. magellanica*, las concentraciones utilizadas en las diluciones respectivas fueron de 1.525, 762,5, 381,25, 190,6, 95,3, 47,6 y 23,8 μg/ml, en tanto que para la mosca *C. vicina* fueron de 2.280, 1.140, 570, 285, 142, 71, y 35,5 μg/ml. Los antibióticos seleccionados contra las bacterias Gram positivas y Gram negativas también se diluyeron en las proporciones seriadas de 1:2.

Por último, se adicionaron 50 μΙ de cada dilución a 50 μΙ de medio con la bacteria seleccionada, para un total de 100 μΙ en cada pozo. Los controles positivo y negativo, y el tiempo y la temperatura de incubación fueron los mismos descritos en el proceso anterior. Las pruebas se practicaron por triplicado.

### *Concentración efectiva 50 (IC*_*50*_ *)*

Para obtener el valor de la IC_50_, se utilizó el logaritmo de las concentraciones de las excreciones y secreciones menores de 10 kDa de las dos especies de moscas frente al porcentaje de inhibición obtenido, con lo que se generó una gráfica (curva dosis-respuesta) cuyos puntos se ajustaron mediante regresión no lineal a una ecuación sigmoidal de cuatro parámetros. A partir de esta ecuación, se calculó la concentración de las excreciones y las secreciones tanto de *C. vicina* como de *S. magellanica*, para obtener una inhibición del 50 % de las bacterias evaluadas.

### Análisis estadísticos

Se construyó una base de datos con los resultados obtenidos y se hizo el análisis estadístico descriptivo correspondiente. Con el programa Stata 12, se hizo un ANOVA de una vía para determinar diferencias entre las excreciones y secreciones nativas, y las mayores y menores de 10 kDa de *C. vicina* y *S. magellanica*, seguido de la prueba *post hoc* de Bonferroni para establecer cuál de las variables estudiadas aportaba dicha diferencia.

Asimismo, con la prueba t de Student se evaluó si se presentaban diferencias significativas entre la actividad antimicrobiana de las excreciones y secreciones nativas, y las menores de 10 kDa de cada una de las especies de mosca estudiadas.

Los datos se analizaron con un índice de confianza del 95 %, en el cual un valor de p<0,05 indicaba diferencias significativas. Para determinar si existían diferencias significativas entre el grado de sensibilidad de las bacterias frente a las excreciones y secreciones menores de 10 kDa de *C. vicina* y *S. magellanica*, se hizo un ANOVA y se practicó la prueba *post hoc* de Bonferroni con los datos obtenidos de las IC50 y una significación de p<0,05.

## Resultados

### Colonización y mantenimiento de la colonia

Los especímenes adultos de *C. vicina* y *S. magellanica* se mantuvieron bajo condiciones controladas de laboratorio; las dos especies se adaptaron a las condiciones físicas, ambientales y nutricionales establecidas. Las larvas de tercer estadio de las dos especies se obtuvieron aproximadamente cinco días después de la oviposición. Hubo continuidad del ciclo de vida de las moscas en varias generaciones, lo cual posibilitó el suministro de material biológico en las pruebas correspondientes.

### Obtención y filtración de excreciones y secreciones larvarias

Para *C. vicina* y *S. magellanica*, se obtuvieron 15 ml de excreciones y secreciones nativas a partir de 3.000 larvas, las cuales tuvieron un peso aproximado de 134,4 g. A partir de 10 ml de las nativas de *C. vicina* y *S. magellanica*, se obtuvieron 6 ml de las menores de 10 kDa y 4 ml de las mayores de 10 kDa, de cada una de las especies evaluadas.

### Cuantificación de proteínas

Las excreciones y secreciones nativas y las menores de 10 kDa de *C. vicina*, registraron una concentración de proteínas de 6,764 μg/ml y 4,561 μg/ ml, respectivamente, mientras que la concentración de las de *S. magellanica* fue menor en comparación con la anterior, teniendo en cuenta que para las nativas fue de 4,674 μg/ml y, para las menores de 10 kDa, de 4,050 μg/ml.

### Actividad antimicrobiana

**Figura 1.**
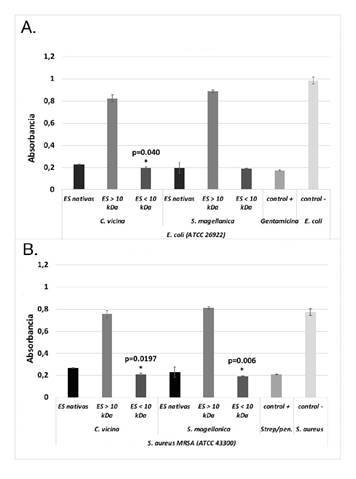
Actividad antimicrobiana de las excreciones y secreciones nativas y de las fracciones obtenidas mediante filtración (mayores y menores de 10 kDa). La sensibilidad bacteriana se evaluó mediante pruebas de turbidimetría. **A.** Actividad antimicrobiana contra *E. coli* (ATCC 26922). **B.** Actividad antimicrobiana contra *S. aureus* (ATCC 43300) * Inhibiciónsignificativa del crecimiento de *E. coli* y *S. aureus*

*Análisis de inhibición de crecimiento en medio líquido mediante turbidimetría*. Al evaluar la actividad antimicrobiana de las excreciones y secreciones nativas, y las mayores y menores de 10 kDa de *C. vicina* contra *S. aureus* MRSA y *E. coli*, se evidenció que las nativas y las menores de 10 kDa registraron una potente actividad antimicrobiana (p=0,000), en tanto que las mayores de 10 kDa no inhibieron el crecimiento de estas bacterias (figura 1, A y B), razón por lo cual esta última fracción no se utilizó en las siguientes pruebas. Además, se encontró que las excreciones y secreciones menores de 10 kDa fueron diferencialmente más efectivas que las nativas en cuanto a los resultados obtenidos con *E. coli* (p=0,040) (figura 1A); sin embargo, en el caso de *S. aureus*, no hubo diferencias entre ellas (p=0,217) (figura 1B). En la especie *S. magellanica*, los hallazgos fueron similares, pero al contrario de *C. vicina*, se determinó que las excreciones y secreciones menores de 10 kDa mostraban diferencias significativas frente a la bacteria *S. aureus* MRSA (p=0,006) (figura 1B), en tanto que no hubo diferencias entre las nativas y las menores de 10 kDa de C. vicina (p=1,000) (figura 1A).

**Figura 2.**
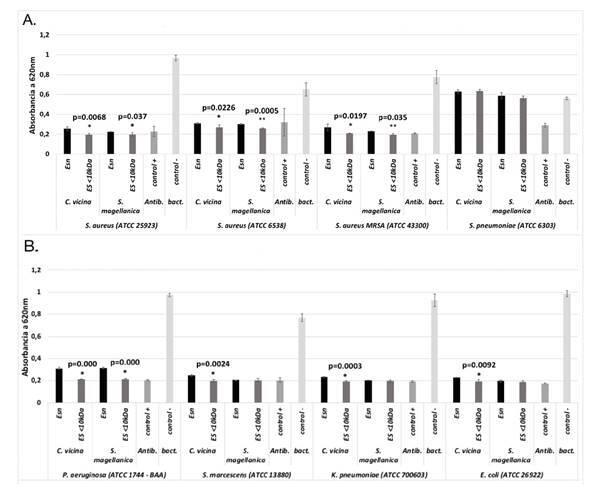
Actividad antimicrobiana de las excreciones y secreciones nativas y de las menores de 10 kDa de *C. vicina* y *S. magellanica*. **A.** Actividad frente a bacterias Gram positivas. **B.** Actividad frente a bacterias Gram positivas. La sensibilidad bacteriana se evaluó mediante pruebas de turbidimetría. * Diferencia significativa: p<0,05 ** Diferencia muy significativa: p<0,001

Las excreciones y secreciones nativas y las menores de 10 kDa de *C. vicina*, fueron eficaces contra las bacterias Gram negativas (figura 2A) y Gram positivas evaluadas, excepto para *S. pneumoniae* ATCC 6303 (figura 2B), siendo las menores de 10 kDa las que mostraron una mayor actividad antibacteriana comparadas con las nativas (p<0,0226). En la especie *S. magellanica*, las nativas y las menores de 10 kDa no tuvieron la capacidad de inhibir el crecimiento de *S. pneumoniae*; sin embargo, sí se registró actividad contra las demás bacterias evaluadas (figuras 2, A y B), siendo la fracción menor de 10 kDa más efectiva que las nativas solo contra las tres cepas de *S. aureus* (p<0,0377) y la de *P. aeruginosa* (p=0,0000). Al comparar la actividad de las menores de 10 kDa de *C. vicina* y *S. magellanica*, no se registraron diferencias significativas en ninguna de las bacterias evaluadas (p>0,2897) (figuras 2, A y B).

*Análisis de la concentración mínima inhibitoria en medio líquido mediante turbidimetría*. Se observó que las concentraciones evaluadas de las excreciones y secreciones menores de 10 kDa, no inhibieron completamente el crecimiento microbiano de ninguna de las bacterias evaluadas; no obstante, los antibióticos utilizados como control positivo tampoco inhibieron el 100 % del crecimiento.

Tanto con *C. vicina* como *S. magellanica*, la primera dilución, correspondiente a una concentración de 2,280 μg/ml y 1,525 μg/ml, respectivamente, fue la más efectiva para inhibir el crecimiento de las bacterias Gram positivas y Gram negativas.

### *Concentración efectiva 50 (IC*_*50*_ *)*

El análisis estadístico evidenció diferencias significativas en la sensibilidad de cada una de las especies bacterianas evaluadas en presencia de las excreciones y secreciones menores de 10 kDa de las dos especies de moscas. En la prueba *post hoc* de Bonferroni, se observó que las de *C. vicina* tuvieron un mayor efecto contra las bacterias Gram negativas, siendo *P. aeruginosa* la que aportaba diferencias significativas comparada con las demás bacterias (p<0,05), en tanto que, para las bacterias Gram positivas, no hubo diferencias (cuadro 1); en cuanto a las de *S. magellanica*, se observó que las diferencias fueron aportadas por bacterias Gram negativas como *K. pneumoniae*, *P. aeruginosa* y *S. marcescens* (p<0,05) y S. aureus ATCC 43300 (p<0,05) (cuadro 2), lo cual indica que las IC_50_ de las excreciones y secreciones menores de 10 kDa de esta especie de mosca tienen un amplio espectro y puso de manifiesto que las menores de 10 kDa de *C. vicina* y *S. magellanica* se comportaron de forma distinta.

**Cuadro 1:** CIM e IC_50_ de la actividad antimicrobiana de las excreciones y secreciones menores de 10 kDa de *C. vicina* frente a diferentes cepas Gram positivas y Gram negativas

*Calliphora vicina*		
Bacteria	CIM μg/ml	IC_50_ μg/ml
*S. aureus* ATCC 6538	2.280	374,11 ± 15,52
*S. aureus* ATCC 25923	2.280	447,86 ± 47,18
*S. aureus* ATCC 43300 MRSA	2.280	830,29 ± 10,69
*E. coli*	2.280	756,39 ± 27,77
*P. aeruginosa	2.280	1537,60 ± 407,41
*K. pneumoniae*	2.280	336,77 ± 21,96
*S. marcescens*	2.280	540,24 ± 57,00

* Diferencia significativa: p<0,05

**Cuadro 2:** CIM e IC_50_ de la actividad antimicrobiana de las excreciones y secreciones menores de 10 kDa de *S. magellanica* frente a diferentes cepas Gram positivas y Gram negativas

Sarconesiopsis magellanica		
Bacteria	CIM μg/ml	lC_50_ μg/ml
*S. aureus* ATCC 6538	1.525	534,50 ± 63,36
*S. aureus* ATCC 25923	1.525	579,38 ± 19,95
* S. aureus ATCC 43300 MRSA	1.525	118,95 ± 118,95
*E. coli*	1.525	503,75 ± 23,93
*P. aeruoginosa	1.525	264,12 ± 264,12
*K. pneumoniae	1.525	320,95 ± 8,56
*S. marcescens	1.525	256,50 ± 27,29

* Diferencia significativa: p<0,05

## Discusión

En el presente estudio se demostró que tanto las excreciones y secreciones nativas, como las menores de10 kDa de *C. vicina* y *S. magellanica*, exhibieron actividad antimicrobiana contra tres bacterias Gram positivas, *S. aureus* ATCC 6538, *S. aureus* ATCC 25923 y S. aureus MRSA ATCC 43300, y contra cuatro bacterias Gram negativas, *E. coli* (ATCC 26922), *P aeruginosa* (ATCC 1744 - BAA), *S. marcescens* (ATCC 13880) y K*. pneumoniae* ATCC (ATCC 700603).

Por otra parte, no se registró actividad contra *S. pneumoniae* ATCC 6303, lo cual podría relacionarse con la protección que ofrece la cápsula de lipopolisacáridos de este microorganismo, el cual sería, probablemente, el factor de virulencia más importante, pues previamente ha exhibido actividad antifagocítica, ha disminuido la autólisis y ha reducido la exposición a los antibióticos ^20^.

En cuanto a los resultados obtenidos a partir de las cepas de S. aureus evaluadas, se observó que el potencial antimicrobiano de las excreciones y secreciones menores de 10 kDa de *C. vicina* y de *S. magellanica*, guardó semejanza con lo hallado en un estudio previo en el que se demostró actividad antibacteriana significativa de la fracción menor de 500 Da aislada de las excreciones y secreciones de *L. sericata* contra un amplio espectro de cepas de *S. aureus* MRSA ^21^.

Sin embargo, la fracción mayor de 10 kDa aislada de *C. vicina* y *S. magellanica* no inhibió el crecimiento bacteriano, lo cual coincide con lo reportado para la fracción mayor de 10 kDa de *L. sericata*^13^, lo que significaría que las moléculas que se encuentran en rangos de bajo peso molecular son las que realmente poseen actividad antimicrobiana; de hecho, en investigaciones previas se ha reportado que los péptidos antimicrobianos, como la lucifencina (aislada y purificada de *L. sericata*), se encuentran en un rango de peso molecular de 0,5 a 10 kDa ^22^.

Además, en el presente estudio se observó inhibición significativa de las excreciones y secreciones nativas contra *S. aureus*, siendo similar a los resultados obtenidos en estudios anteriores en los que se observó una potente actividad de las de *S. magellanica* contra diferentes cepas de *S. aureus* a partir de las pruebas de turbidimetría ^15^.

Contra las bacterias Gram negativas, la duración y potencia de la actividad antibacteriana fue diferente a lo reportado en algunos estudios previos. En el presente trabajo, se observó que las excreciones y secreciones nativas y las menores de 10 kDa de *C. vicina* y *S. magellanica*, exhibieron actividad contra *P. aeruginosa* hasta las 18 horas de evaluación, en tanto que, en un estudio previo, se reportó que, en la prueba de unidades formadoras de colonia (UFC), las excreciones y secreciones nativas de la especie *C. vicina* inicialmente redujeron el número de colonias, pero después de 8 horas nuevamente hubo crecimiento de la bacteria ^16^.

Por otro lado, las excreciones y secreciones nativas y las menores de 10 kDa, inhibieron significativamente el crecimiento de *S. marcescens* durante 18 horas, pero en otro estudio se reportó que las nativas de *C. megacephala* y *C. putoria* solo mantuvieron su potencial antimicrobiano durante las primeras 6 horas de las 22 horas de estudio ^17^.

Por último, las excreciones y secreciones nativas y las menores de 10 kDa tanto de *C. vicina* como de *S. magellanica*, presentaron actividad contra *K. pneumoniae*, en tanto que las nativas de *L. sericata* estudiadas no inhibieron la bacteria en la prueba de difusión en disco ^14^. En cuanto a *E. coli*, en la prueba de UFC, en la de difusión en disco y en la turbidimetría de los estudios ya mencionados, los resultados fueron similares, ya que las excreciones y secreciones evaluadas demostraron potencial actividad antimicrobiana independientemente de la especie de mosca estudiada ^14^^,^^16^^,^^17^.

Los resultados del presente estudio que no concuerdan con investigaciones previas ^14^^,^^16^^,^^17^ podrían explicarse al considerar otras variables, por ejemplo, las diferentes técnicas utilizadas para evaluar la actividad antimicrobiana: en el presente estudio, se evaluó la actividad antibacteriana mediante turbidimetría porque se considera que tiene mayor sensibilidad que otras técnicas frecuentemente usadas. Otros posibles factores que pudieron influir en los resultados incluyen el número de larvas utilizadas y el pretratamiento con las cepas bacterianas en las larvas, el cual pudo haber aumentado la concentración de moléculas con propiedades antibacterianas de las excreciones y secreciones larvarias ^15^.

En los ensayos de CIM se observó que las excreciones y secreciones menores de 10 kDa de las dos especies de moscas evaluadas exhibieron actividad antimicrobiana frente a las bacterias Gram positivas y Gram negativas. En la IC_50_ se encontraron algunas diferencias en la inhibición del crecimiento de las bacterias, evidenciándose que las excreciones y secreciones menores a 10 kDa de *S. magellanica* tenían un amplio espectro, en tanto que las de *C. vicina* tuvieron una mejor actividad contra las bacterias Gram negativas. En estudios previos, se ha sugerido que las bacterias Gram positivas son más sensibles a las excreciones y secreciones larvarias y que las bacterias Gram negativas requieren mayores concentraciones para su inhibición ^23^^,^^24^, aunque algunos autores sugieren lo contrario ^16^^,^^17^, por lo que son necesarios más estudios que conduzcan a resultados concluyentes.

Por otro lado, a pesar de que las excreciones y secreciones menores de 10 kDa de *C. vicina* y *S. magellanica* presentaron una potente actividad antimicrobiana, esta actividad no fue bactericida; es por esto que la combinación de excreciones y secreciones larvarias con agentes antimicrobianos podría tener un mayor potencial terapéutico ^24^^,^^25^ y constituirse en una alternativa para el tratamiento de infecciones causadas por bacterias resistentes a los antibióticos ^26^. Es importante resaltar que la combinación sinérgica de las excreciones y secreciones con los antibióticos puede retrasar el desarrollo de mecanismos de resistencia ^27^.

Además, en algunos de los componentes antimicrobianos de las excreciones y secreciones larvarias se han registrado compuestos alcalinos, como carbonato de amonio, calcio, alantoína y urea, los cuales tienen acción sobre el crecimiento microbiano ^11^. Además, metaloproteinasas de matriz (MMP) como la quimiotripsina, tienen efecto inhibitorio sobre la formación de biopelículas, y la desoxirribonucleasa (ADNsa) impide tanto el crecimiento bacteriano como la formación de biopelículas ^7^.

Por otro lado, los péptidos antimicrobianos, importantes componentes de las excreciones y secreciones larvarias, actúan como mecanismo de defensa del huésped, poseen un potente poder bactericida y tienen la capacidad de neutralizar toxinas ^28^. En estudios previos, se han aislado, caracterizado y evaluado dos péptidos antimicrobianos de las excreciones y secreciones larvarias de *S. magellanica*, los cuales demostraron una potente actividad antimicrobiana ^29^^,^^30^. No obstante, es importante continuar estudiando las propiedades de las excreciones y secreciones larvarias de *S. magellanica* y *C. vicina*, puesto que podrían ser útiles por separado o de forma sinérgica para el desarrollo de potenciales fármacos o para la producción de nuevos agentes antiinfecciosos que, entre otras funciones, tendrían aplicación terapéutica tópica en heridas crónicas, por ejemplo, aquellas asociadas a ulceras diabéticas. Por ello, la purificación y la producción en masa de tales péptidos son de máxima prioridad.

Se ha evidenciado que, además de su potente poder antimicrobiano, las excreciones y secreciones larvarias de los califóridos también tienen actividad antifúngica ^31^^,^^32^, antiparasitaria, antiinflamatoria ^33^, y procoagulante ^34^^,^^35^, lo cual aumenta positivamente el interés en estas especies de moscas.

Todos estos hallazgos sugieren que las propiedades de los componentes de las excreciones y secreciones larvarias derivadas de estas especies de moscas necrófagas son potencialmente promisorias para el aislamiento y desarrollo de agentes antimicrobianos. En resumen, se demostró que las excreciones y secreciones nativas y las menores de 10 kDa de larvas de las especies *C. vicina* y *S. magellanica*, exhibieron una potente actividad antibacteriana contra tres cepas de *S. aureus* y cuatro bacterias Gram negativas, siendo las excreciones y secreciones menores de10 kDa más efectivas que las nativas en las dos especies de moscas evaluadas. Las excreciones y secreciones menores de 10 kDa presentaron la misma efectividad, excepto cuando se determinó la IC_50_ pues se observó que las excreciones y secreciones de la especie *S. magellanica* tenían un amplio espectro de acción, en tanto que las de *C. vicina* tenían un mayor potencial frente a bacterias Gram negativas.
